# Supplementary material for: Transcriptional and Translational Dynamics of Zika and Dengue Virus Infection
Source: Viruses. 2022 Jun 28;14(7):1418. doi: 10.3390/v14071418 (PMC9316442; doi:10.3390/v14071418)
Supplement: Supplementary file 1 [file viruses-14-01418-s001.zip › viruses-1746562-supplementary.pdf]

Figure S1

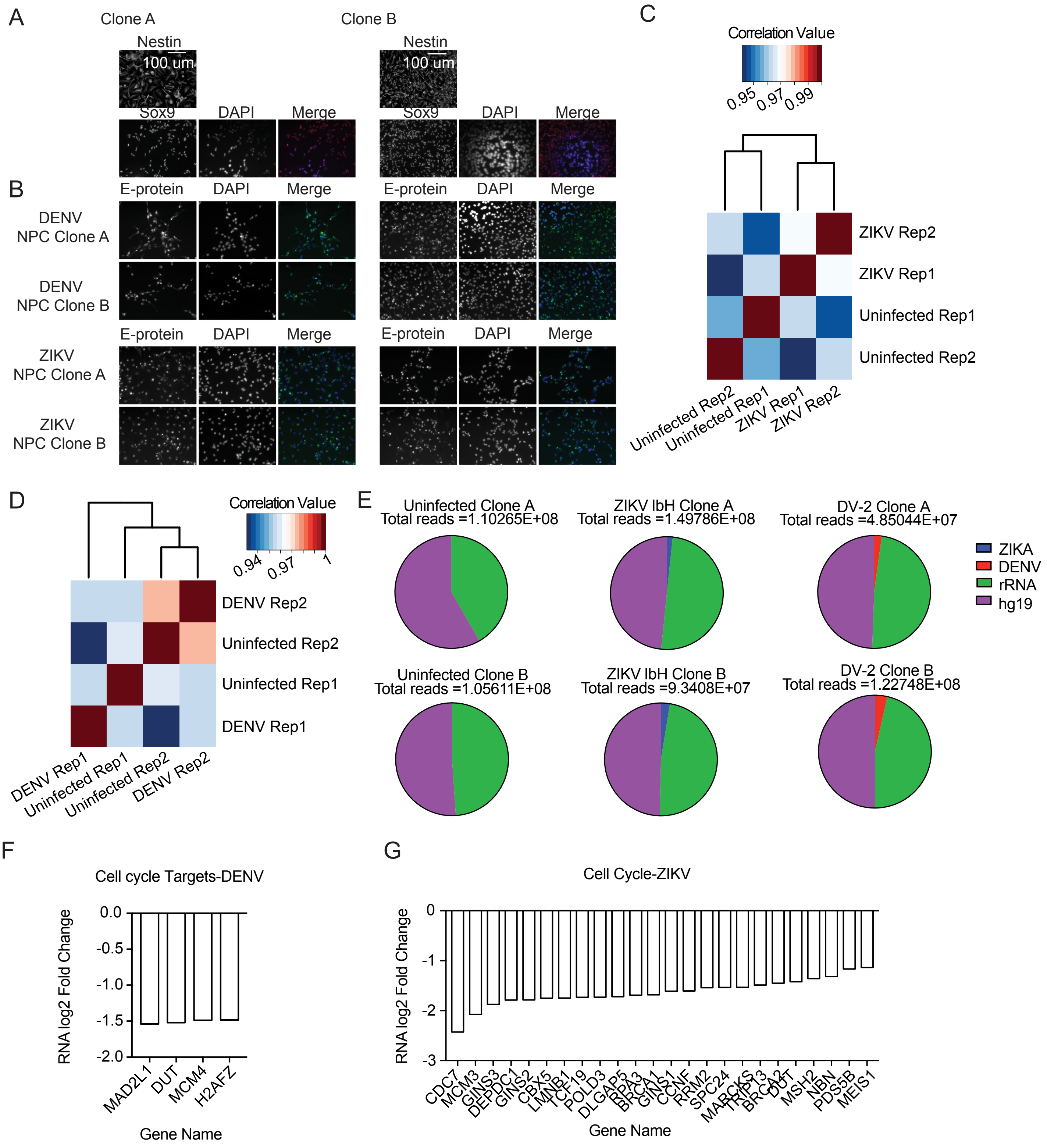
A
Clone A
Clone B
Nestin
100 um
Sox9
DAPI
Merge
B
E-protein
DAPI
Merge
DENV
NPC Clone A
DENV
NPC Clone B
ZIKV
NPC Clone A
ZIKV
NPC Clone B
C
Correlation Value
0.95
0.97
0.99
ZIKV Rep2
ZIKV Rep1
Uninfected Rep1
Uninfected Rep2
D
Correlation Value
0.94
0.97
1
DENV Rep2
Uninfected Rep2
Uninfected Rep1
DENV Rep1
E
Uninfected Clone A
Total reads =1.10265E+08
ZIKV IbH Clone A
Total reads =1.49786E+08
DV-2 Clone A
Total reads =4.85044E+07
Uninfected Clone B
Total reads =1.05611E+08
ZIKV IbH Clone B
Total reads =9.3408E+07
DV-2 Clone B
Total reads =1.22748E+08
ZIKA
DENV
rRNA
hg19
F
Cell cycle Targets-DENV
RNA log2 Fold Change
0.0
-0.5
-1.0
-1.5
-2.0
MAD2L1
DUT
MCM4
H2AFZ
Gene Name
G
Cell Cycle-ZIKV
RNA log2 Fold Change
0
-1
-2
-3
CDC7
MCM3
GINS3
DEPDC1
GINS2
CBX5
LMNB1
TCF19
POLD3
DLGAP5
RPA3
BRCA1
GINS1
CCNF
RRM2
SPC24
MARCKS
TRIP13
BRCA2
DUT
MSH2
NBN
PDS5B
MEIS1
Gene Name

Figure S2

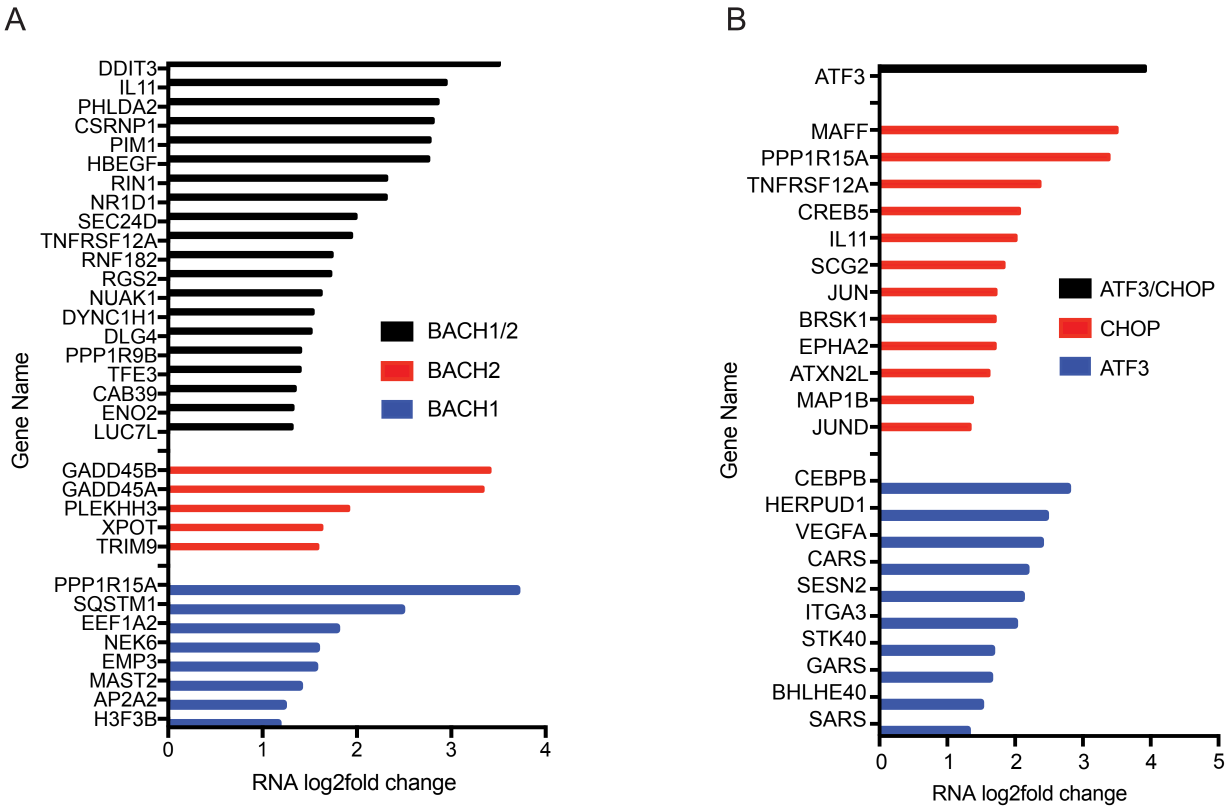
A
DDIT3
IL11
PHLDA2
CSRNP1
PIM1
HBEGF
RIN1
NR1D1
SEC24D
TNFRSF12A
RNF182
RGS2
NUAK1
DYNC1H1
DLG4
PPP1R9B
TFE3
CAB39
ENO2
LUC7L
GADD45B
GADD45A
PLEKHH3
XPOT
TRIM9
PPP1R15A
SQSTM1
EEF1A2
NEK6
EMP3
MAST2
AP2A2
H3F3B
Gene Name
0
1
2
3
4
RNA log2fold change
BACH1/2
BACH2
BACH1
B
ATF3
MAFF
PPP1R15A
TNFRSF12A
CREB5
IL11
SCG2
JUN
BRSK1
EPHA2
ATXN2L
MAP1B
JUND
CEBPB
HERPUD1
VEGFA
CARS
SESN2
ITGA3
STK40
GARS
BHLHE40
SARS
Gene Name
0
1
2
3
4
5
RNA log2fold change
ATF3/CHOP
CHOP
ATF3

Figure S3

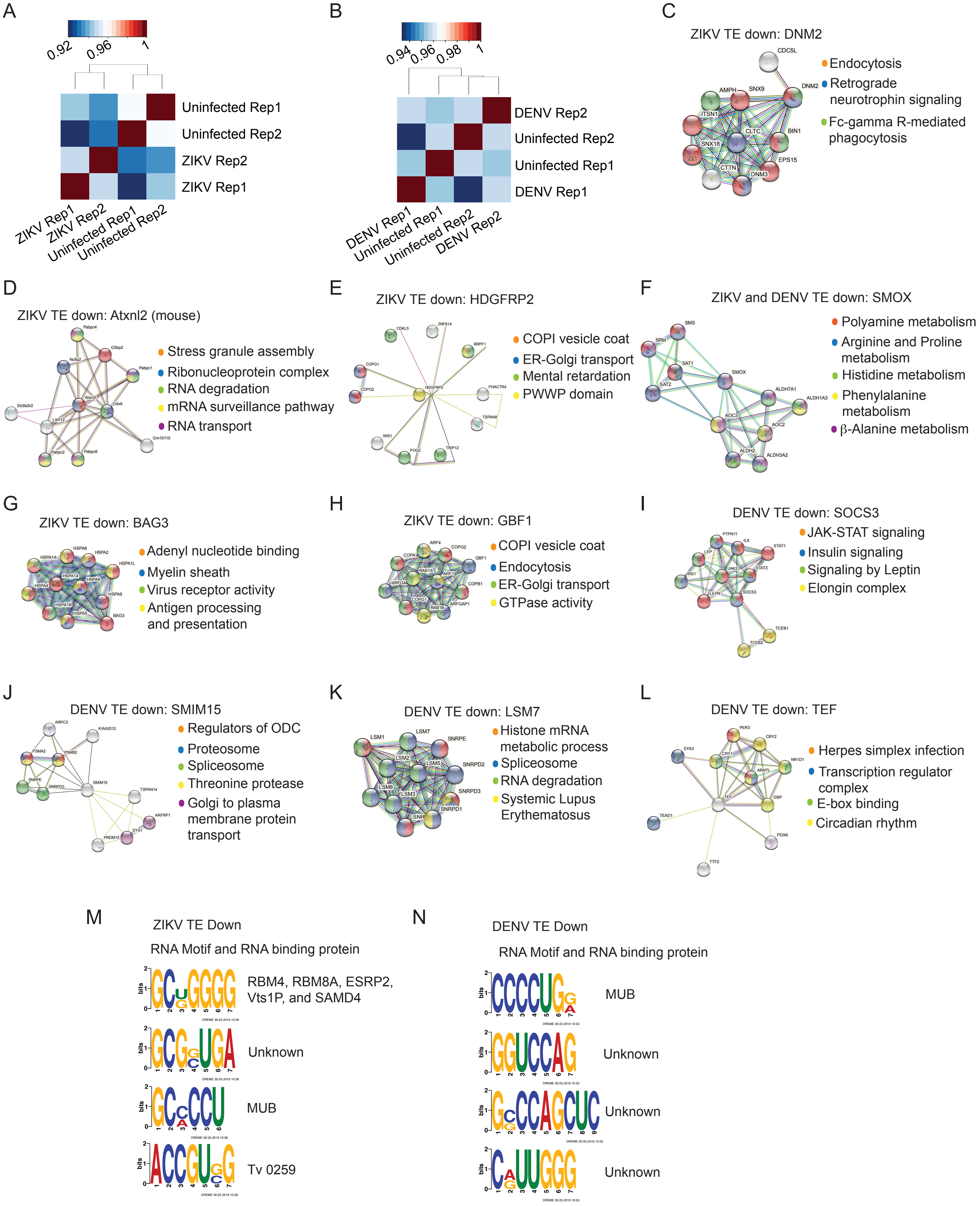

A
0.92
0.96
1
Uninfected Rep1
Uninfected Rep2
ZIKV Rep2
ZIKV Rep1
ZIKV Rep1
ZIKV Rep2
Uninfected Rep1
Uninfected Rep2
B
0.94
0.96
0.98
1
DENV Rep2
Uninfected Rep2
Uninfected Rep1
DENV Rep1
DENV Rep1
Uninfected Rep1
Uninfected Rep2
DENV Rep2
C
ZIKV TE down: DNM2
Endocytosis
Retrograde neurotrophin signaling
Fc-gamma R-mediated phagocytosis
D
ZIKV TE down: Atxnl2 (mouse)
Stress granule assembly
Ribonucleoprotein complex
RNA degradation
mRNA surveillance pathway
RNA transport
E
ZIKV TE down: HDGFRP2
COPI vesicle coat
ER-Golgi transport
Mental retardation
PWWP domain
F
ZIKV and DENV TE down: SMOX
Polyamine metabolism
Arginine and Proline metabolism
Histidine metabolism
Phenylalanine metabolism
β-Alanine metabolism
G
ZIKV TE down: BAG3
Adenyl nucleotide binding
Myelin sheath
Virus receptor activity
Antigen processing and presentation
H
ZIKV TE down: GBF1
COPI vesicle coat
Endocytosis
ER-Golgi transport
GTPase activity
I
DENV TE down: SOCS3
JAK-STAT signaling
Insulin signaling
Signaling by Leptin
Elongin complex
J
DENV TE down: SMIM15
Regulators of ODC
Proteosome
Spliceosome
Threonine protease
Golgi to plasma membrane protein transport
K
DENV TE down: LSM7
Histone mRNA metabolic process
Spliceosome
RNA degradation
Systemic Lupus Erythematosus
L
DENV TE down: TEF
Herpes simplex infection
Transcription regulator complex
E-box binding
Circadian rhythm
M
ZIKV TE Down
RNA Motif and RNA binding protein
RBM4, RBM8A, ESRP2, Vts1P, and SAMD4
Unknown
MUB
Tv 0259
N
DENV TE Down
RNA Motif and RNA binding protein
MUB
Unknown
Unknown
Unknown

Figure S4

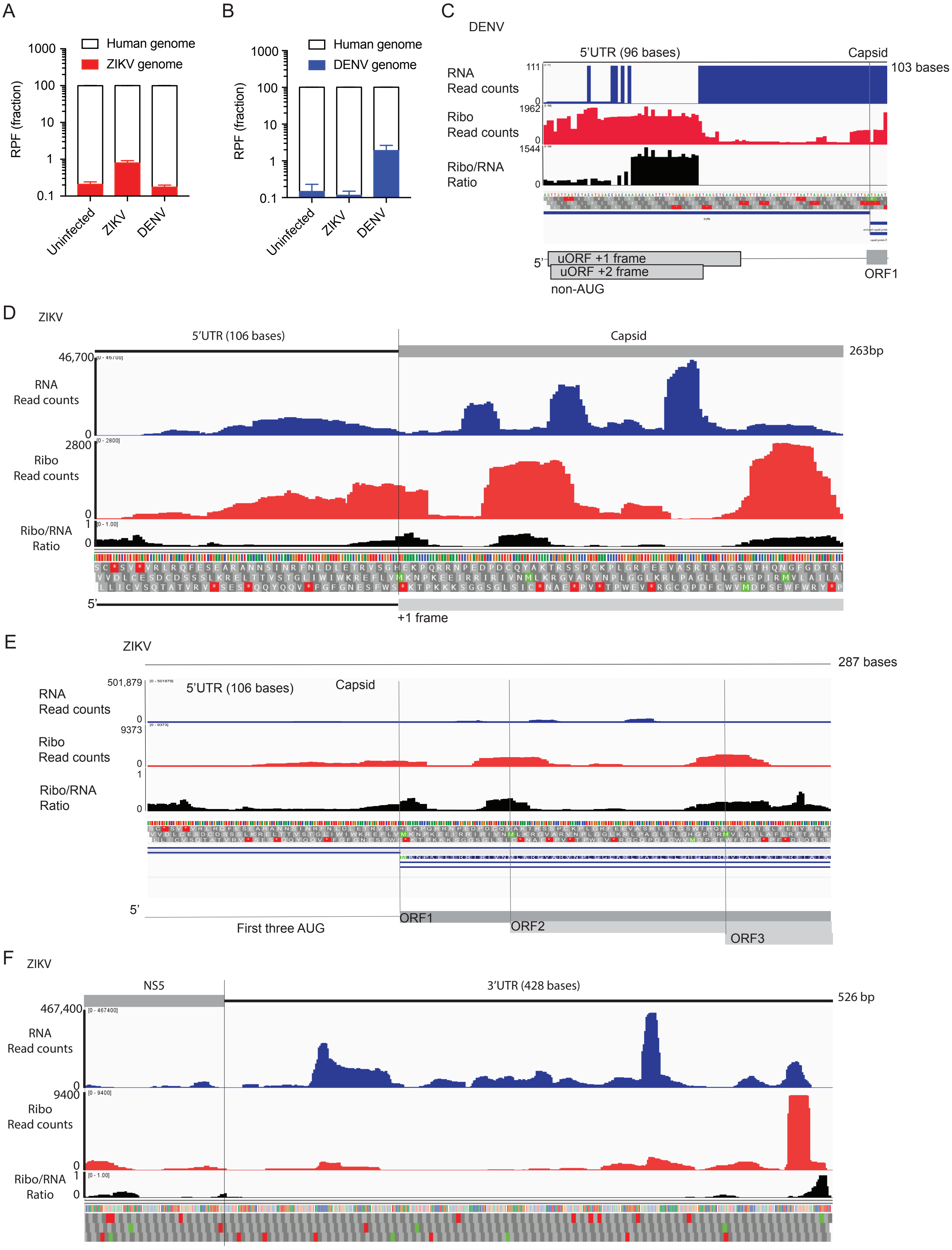

A
1000
100
10
1
0.1
RPF (fraction)
Human genome
ZIKV genome
Uninfected
ZIKV
DENV
B
1000
100
10
1
0.1
RPF (fraction)
Human genome
DENV genome
Uninfected
ZIKV
DENV
C
DENV
5'UTR (96 bases)
Capsid
103 bases
RNA Read counts
111
0
Ribo Read counts
1962
0
Ribo/RNA Ratio
1544
0
5'
uORF +1 frame
uORF +2 frame
non-AUG
ORF1
D
ZIKV
5'UTR (106 bases)
Capsid
263bp
46,700
RNA Read counts
0
2800
Ribo Read counts
0
1
Ribo/RNA Ratio
0
5'
+1 frame
E
ZIKV
287 bases
501,879
RNA Read counts
0
9373
Ribo Read counts
0
1
Ribo/RNA Ratio
0
5'UTR (106 bases)
Capsid
5'
First three AUG
ORF1
ORF2
ORF3
F
ZIKV
NS5
3'UTR (428 bases)
526 bp
467,400
RNA Read counts
0
9400
Ribo Read counts
0
1
Ribo/RNA Ratio
0
